# The Internationalisation of Tobacco Control, 1950–2010

**DOI:** 10.1017/mdh.2016.97

**Published:** 2016-10

**Authors:** David Reubi, Virginia Berridge

**Affiliations:** 1 Department of Global Health & Social Medicine, King’s College London, Strand,London WC2R 2LS, UK; 2 Centre for History in Public Health, London School of Hygiene and Tropical Medicine, 15–17 Tavistock Place, London WC1H 9SH, UK

**Keywords:** International health, Global health, Tobacco control, Smoking, Expert and advocacy networks, WHO Framework Convention on Tobacco Control

## Abstract

This article explores the internationalisation of tobacco control as a case study in the history of international health regulation. Contrary to the existing literature on the topic, it argues that the history of international anti-smoking efforts is longer and richer than the making of the World Health Organisation’s Framework Convention on Tobacco Control in the early twenty-first century. It thereby echoes the point made by other scholars about the importance of history when making sense of contemporary global health. Specifically, the article shows how the internationalisation of tobacco control started in the 1950s through informal contacts between scientists working on cancer research and how these initial interactions were followed by a growing number of more formal initiatives, from the World Conferences on Tobacco or Health to the Bloomberg Initiative to Reduce Tobacco Use. Rather than arranging these efforts in a linear narrative of progress culminating with the Framework Convention on Tobacco Control, we take anthropological claims about global health’s uneven terrain seriously and portray a history of international tobacco control marked by ruptures and discontinuities. Specifically, we identify three successive periods, with each of them characterised by specific understandings of international action, tobacco control expertise, advocacy networks and funding strategies.

## 1 Introduction

This article explores the internationalisation of tobacco control in historical perspective.^1^ In most of the existing historical, public health and social science literature, this process is predominantly associated with the making of the World Health Organisation’s (WHO) Framework Convention on Tobacco Control (FCTC) at the start of the twenty-first century.^2^ If other international anti-smoking efforts are mentioned at all, they are usually cast as early, minor developments in a Whiggish history culminating with the adoption of the FCTC in 2003. In some respect, this fascination with the FCTC is understandable. Indeed, the FCTC was not just the first international health treaty negotiated under the auspices of the WHO, but was also the first international treaty on tobacco control to be ever adopted.^3^ Furthermore, the FCTC was only made possible by the pioneering efforts of a transnational coalition of civil society organisations and has led to the creation of two, new supranational institutions in charge of its implementation.^4^ But, there are also inherent difficulties with the standard narrative and the way it reduces the internationalisation of tobacco control to the making of the FCTC. To start with, it fails to account for most of the other international anti-smoking efforts that either pre-dated or happened at the same time as the FCTC.^5^ And when it does account for some of these other efforts, it assumes that they necessarily share the same ideas about international action and tobacco control as the FCTC, thereby suppressing any difference, contestation or rupture in the internationalisation of tobacco control.^6^

In this article, we challenge and complexify the standard narrative on the internationalisation of tobacco control found in the literature. While we do not dispute the importance of the FCTC in international tobacco control, we suggest that the internationalisation of anti-smoking efforts has a much longer and richer history than the making of the WHO treaty, echoing thereby a point made by other scholars about the importance of history when making sense of the contemporary global health landscape.^7^ Specifically, we show how there has been multiple international collaborative efforts in the field of tobacco control over the last sixty years, from informal contacts between scientists working on cancer and the organisation of the first World Conferences on Tobacco or Health (hereafter: World Conferences)^8^ in the 1950s and 1960s to the World Bank’s work on tobacco control economics and the launch of the *Bloomberg Initiative to Reduce Tobacco Use* in the 2000s. Rather than arranging these efforts in a linear narrative of progress culminating with the FCTC, we take anthropological claims about global health’s uneven and contested terrain seriously and depict a history of international tobacco control marked by ruptures and shifts.^9^ In particular, we suggest that this history is made of three successive periods, with each period characterised by specific understandings of international action, knowledge about tobacco control, structures of financial support and networks of experts and advocates.

## 2 Methodology

Before examining the history of international tobacco control efforts, we briefly outline our historical research methods. To start with, all the relevant articles were gathered from a literature search on the internationalisation of tobacco control.^10^ The search was conducted using a range of keywords (e.g. international tobacco control and World Conference) in online databases (e.g. PubMed and JStore). The resources of major libraries such as the British and Wellcome Libraries and that of the WHO Headquarters, the London School of Hygiene and Tropical Medicine and the American Cancer Society (ACS) were also used. Similarly, archival collections, such as the Action for Smoking and Health archive at Wellcome, were utilised. Relevant online documents (reports, pamphlets, newsletters, conference proceedings etc.) were also obtained from organisations active in the internationalisation of tobacco control, including: the WHO; the World Conferences; the International Union against Cancer (UICC); the ACS; GlobaLink; the World Bank; the Campaign for Tobacco Free Kids (CTFK); International Union against Tuberculosis and Lung Diseases (UITLD): Bloomberg Philanthropies; the Bill and Melinda Gates Foundation; the International Development Research Council (IDRC); the Framework Convention Alliance (FCA); and the Rockefeller Foundation. Furthermore, in-depth, semi-structured interviews were conducted with over eighty experts and advocates from the Americas, Asia, Africa and Europe active in the internationalisation of tobacco control. In addition, the authors participated in a Witness Seminar on the FCTC jointly organised by the WHO Global Health Histories Project and the Wellcome Trust History of Modern Biomedicine Research Group in February 2010.^11^ The articles, documents and interviews, among other things, were examined in detail to: identify the main institutions and actors involved; understand the development of international action in the field of tobacco control; determine the different understandings of international action at work; ascertain the networks, resources and knowledge that made international action possible; and examine how all these had changed over time.

## 3 Sixty Years of International Collaborative Efforts

In the late 1940s, when Austin Bradford Hill and Richard Doll were exploring the links between smoking and cancer at the London School of Hygiene and Tropical Medicine, they had been unaware of the parallel study being carried out by American researchers Ernst Wynder and Evarts Graham, which was published just before theirs.^12^ The first international contacts that did develop in the post war years were Anglo-American and the result of individual, informal initiatives.^13^ Bradford Hill, for example, gave a presentation of his work in a Harvard lecture in the 1950s. Similarly, Wynder visited the British Ministry of Health in 1953, where officials were doubtful about his scientific objectivity.^14^ Some years later, British doctor Charles Fletcher, secretary of the Royal College of Physicians’ (RCP) Committee on Smoking, met American epidemiologist Daniel Horn and others to discuss the idea of a World Conference.^15^

### 3.1 Stage 1: International Conferences and Exchanges, Mid-1960s to Late 1970s

These initial, informal contacts led to a wave of more formal and sustained international efforts between the mid-1960s and the late 1970s. This, of course, was a period when, following the publication of reports on smoking and health by national medical authorities such as the RCP and the US Surgeon General, there was a growing public awareness about the dangers of tobacco in the Western world.^16^ It was also a time when medicine and public health in advanced industrialised societies were being reconfigured around chronic diseases and unhealthy lifestyles.^17^ The most significant international tobacco control initiative during this period was the organisation, led by the ACS, of the first three World Conferences. Held in New York in 1967, in London in 1971 and again in New York in 1975, they were critical in bringing the emergent international tobacco control field together at this early stage.^18^ The conferences were followed by two more important initiatives. The first was the WHO’s recognition of smoking as a critical issue in the 1970s.^19^ Working through the organisation’s Regional Committees, European and North American public health experts succeeded in getting the WHO to issue a series of resolutions and two reports acknowledging the dangers of tobacco use and calling for action by member states.^20^ Interestingly, this was not without some opposition from representatives of developing countries, who argued that there were ‘more important things’ for the WHO to do, such as ‘malaria eradication’ and ‘family planning’.^21^ The second initiative was the development of the UICC’s Smoking and Lung Cancer Programme. Led by Australian physician Nigel Gray with the support of ACS, the programme sought to ‘centralise and publish useful information’ about smoking for national cancer societies, as with its 1977 *Guidelines for Smoking Control*.^22^

During this initial period, international action was conceived as tobacco control experts from individual countries coming together to exchange information about the latest findings, share their experiences and support each other’s national efforts to stymie the smoking epidemic. As George Godber, the British Chief Medical Officer (CMO) and an important figure in the early internationalisation of tobacco control noted in 1971:

We need a collective resolve, a serious attempt to help each other …Where does international action come into this? Just as any ground gained [in one’s own country] makes the next step easier, so does any effective action in another country. The [USA have] helped us all. The action taken in …Ireland has its overtones in Britain. The moves in Canada and Norway will be closely watched by the rest of us …We all need to know the moves of the others, for we are all engaged in closing the same ring …I believe that [the] international exchanges we have …[will be] of the greatest assistance to us all individually.^23^

The World Conferences are illustrative of this particular understanding of international action. This type of conference has a long history in the field of international health, being common since the international health conferences on cholera in the mid-nineteenth century.^24^ Building on these precedents, the first three World Conferences brought together about five hundred epidemiologists, medical scientists, health educators, activists and government officials from the rich industrialised countries of the North such as Australia, Canada, Norway, Sweden, the UK and the USA.^25^ In the words of the organisers, these events were a unique opportunity ‘to compare experiences and to exchange ideas, to report on new medical and scientific findings and to recommend programmes of research, education and public and citizen action against cigarette smoking’.^26^ Put differently, their aim was to enable ‘the interchange of information’ between delegates and ‘stimulate’ them to take ‘action’ against smoking in their own countries.^27^ This was also the aim of *World Smoking & Health*, the World Conference’s *de facto* newsletter that ACS began to publish after the 1975 meeting to allow delegates to communicate about developments in the field and share their experiences ‘at more frequent intervals’.^28^

As a space to exchange information and share experiences, the World Conferences were critical in allowing a nascent ‘worldwide movement against smoking’ to develop.^29^ Indeed, it offered anti-smoking experts and advocates a regular meeting ground where they were able to develop a sense of community and a collective identity articulated around the shared belief that smoking was harmful and had to be curbed.^30^ As one advocate who attended these conferences explained:

The conferences were the chance to meet all these people in the world working on tobacco control. It was a sort of bonding as an international community. The coming together of people from different backgrounds because we knew that smoking was harmful and we all wanted to reduce that harm …The conferences allowed the building of a very strong international community …a brotherhood.^31^

It is noteworthy that most experts that were part of this emerging world movement against tobacco during the 1960s and 1970s were physicians and epidemiologists who were actively involved in problematising and calling for action against smoking in their own countries. Also remarkable was the fact that all these experts came from advanced industrialised countries – a reflection of the way the smoking epidemic had unfolded geographically, starting in developed societies before spreading to the rest of the world.^32^ Examples of some of the more prominent of these experts would include, in addition to the figures already mentioned above: the American Surgeon General Luther Terry; British doctor Keith Balls, a co-founder of Action on Smoking and Health UK; Dr Lars Ramstrom, head of the Swedish National Smoking and Health Association; and Kjell Bjartveit, a Norwegian physician and director of the National Council on Smoking and Health in Oslo.

Most international anti-smoking efforts in the 1960s and 1970s were characterised by a concern with action. Indeed, the experts that made up the budding world movement against smoking were less interested in stressing the dangers of tobacco use for human health than in devising strategies to curb the smoking epidemic. As Terry declared in his opening speech at the 1967 World Conference:

We have come to the end of one era …The period of uncertainty is over …There is no longer any doubt that …smoking is a …threat to …health …Today…we are on the threshold of a new era, a time of action, a time …to work together to bring [smoking] under control.^33^

This new concern with action was also perceptible in what Lars Ramstrom described as the ‘increasingly action-oriented nature’ of the first World Conferences.^34^ Indeed, while the conferences always included some biomedical and epidemiological papers on the relation between smoking and health, most interventions were devoted to developing strategies to fight the smoking epidemic.^35^ Similarly, in contrast to its first report on *Smoking and its Effect on Health*, the WHO’s second report on *Controlling the Smoking Epidemic* announced it was ‘time for action’ and proposed ‘guidelines to control the epidemic’.^36^ This same desire for action also informed the UICC’s work and, specifically, its 1977 *Guidelines for Smoking Control*, as one of the authors explained:

*Guidelines* was the first international publication that talked about what to do about smoking. Most publications until then talked about how bad it was. And we got sick of hearing people talk about how bad it was and not what they could do about it.^37^

For the most part, the strategies for action discussed at the World Conferences and outlined in both the WHO and UICC reports fall into one of four categories. First, there were public information and education programmes, which experts at the time viewed as ‘the most important activity to undertake against smoking’.^38^ Second, there was legislative action and, in particular, legislation on advertising bans and health warnings.^39^ Third, there were low-tar cigarettes, which often generated anxious debates about whether encouraging ‘less dangerous forms of smoking’ did not mislead the public into thinking that smoking was safe after all.^40^ Fourth, there were smoking cessation activities, with experts discussing ‘how to conduct withdrawal clinics [and] the kind of appeal and guidance that is effective’.^41^

As Keck and Sikkink have argued, international action is expensive because of the greater geographical distances and number of languages and cultures involved.^42^ This was also the case with cross-national tobacco control efforts in the 1960s and 1970s. What is remarkable perhaps is that the funds and infrastructure that supported these efforts all came from national rather than international organisations. To start with, there was no full-time paid position dedicated to tobacco control at any international organisation. All experts involved internationally were employed by national organisations such as government agencies and anti-smoking associations. So, for example, Daniel Horn worked for the American National Interagency Council on Smoking and Health while Nigel Gray was hired by the Anti-Cancer Council of Victoria and Lars Ramstrom was head of the Swedish National Smoking and Health Association. Their international work was often done in addition to their national full-time employment and on a voluntary basis.^43^ Likewise, the funds to organise international events and pay for such things as transport, accommodation and catering also came from national organisations. By far the biggest contributors during this early period were the Americans and, in particular, ACS. They financed the first three World Conferences and, together with the Norwegian Government and the Anti-Cancer League of Victoria, paid for the UICC Smoking and Lung Cancer Programme.^44^ As one European advocate reminisced:

The ACS was the large animal in the jungle and incredibly generous. They organised and paid for the World Conferences. They paid for everybody to come. I went in 1975 and the Americans paid for my plane ticket and hotel. They also funded most of the UICC work. They were the biggest funders.^45^

### 3.2 Stage 2: Smoking and the Third World, Late 1970s to Early 1990s

The period from the late 1970s to the early 1990s saw the consolidation and intensification of the international efforts initiated in the preceding stage. The World Conferences, which were held in Stockholm in 1979, Winnipeg in 1983, Tokyo in 1987, Perth in 1990 and Buenos Aires in 1992, are a case in point.^46^ Like their predecessors, these conferences were key meeting places for experts working in the field wanting to share their experiences and learn from each other.^47^ But, they were now run more frequently, increasingly took place outside North America and Europe, drew twice as many participants and were managed more rationally through an international coordination committee that comprised the UICC, the WHO and the UITLD.^48^ While smoking remained a minor concern for the WHO up to the mid-1990s, the Geneva-based organisation also expanded its tobacco control efforts during this second period, with its Director-General Halfdan Mahler explicitly integrating smoking within the primary health care agenda developed at Alma Ata and in the *Health for All by Year* *2000* report.^49^ An important part of these expanded efforts was the establishment of the WHO’s first permanent programme in 1980: the Tobacco or Health Programme.^50^ Even though it was, for a long time, a very limited operation, with an insignificant budget and only one or two members of staff,^51^ the programme oversaw some important developments, including: the preparation of two expert reports on smoking in developing countries;^52^ the organisation of workshops on tobacco control in Asia and Africa; the publication of a few studies on smoking;^53^ and the creation of World No Tobacco Day.^54^

Another aspect of the WHO’s expanded tobacco control efforts was the increasing involvement of its Regional Offices, which organised workshops and drew up action plans for their regions.^55^ Finally, this second period also saw a marked ‘step up’ of the UICC Smoking and Lung Cancer Programme, which became the most important initiative in the field during the 1980s.^56^ Besides the publication of influential anti-smoking manuals, the programme’s key contribution was the organisation of over seventy-five workshops on tobacco control in more than fifty countries across Africa, Latin America, Asia and, after the fall of the Berlin Wall, Eastern Europe and the ex-Soviet Union.^57^

This second stage was marked by a shift in the meaning of international action, with the term increasingly understood through the lens of development. The earlier notion of international action as cross-national information sharing and support did not disappear, of course. But, it came to sit alongside the now dominant view that international action was about developed societies using their tobacco control knowledge to help nations in what was then termed ‘the Third World’ to build their capacity to fight against the smoking epidemic.^58^ This new concept of international action was made possible by two, interrelated developments. The first was the problematisation of smoking in the developing world. For a long time, most public health experts had viewed chronic diseases and contributing risk factors such as smoking as a problem that was exclusive to rich, industrialised countries in the West. Few of them imagined that smoking and related diseases such as cancer could be a problem for developing societies. Indeed, the Third World’s major concerns, they thought, were infectious diseases, child and maternal health issues as well as malnutrition.^59^ From the late 1970s onwards, these views began to shift with tobacco progressively recognised as a problem for developing countries. This shift was part of a broader effort to re-frame chronic disease as a development issue, from the launch in the mid-1980s of the Interhealth programme – the WHO’s first, rather limited, attempt to address chronic diseases in the Third World – to the World Bank’s major push to have chronic diseases recognised as one of the most important health issues in low- and middle-income countries (LMICs) in the early 1990s as part of its Health Sector Priorities Review and Global Burden of Disease projects.^60^ A significant moment in the problematisation of smoking in developing societies was the publication in the late 1970s of *Tobacco and the Third World* and *Cigarette Marketing in the Third World* by two development experts: Mike Muller, a South African who had played a critical role in campaigns against Nestlé’s promotion of artificial baby milk in developing countries, and Bo Wickstrom, a Swedish economist who had worked on food and nutrition in East Africa.^61^ These two publications made clear that, because of Western tobacco companies’ aggressive efforts to create new markets for cigarettes in developing countries, the smoking epidemic was mounting in the Third World. Tobacco control experts were quick to pick up on Muller and Wickstrom’s work and recognise that smoking had become a problem for developing societies. The 1979 World Conference, where Wickstrom came to present his findings,^62^ seemed to have been a turning point in that respect.^63^ As British activist Mike Daube explained in his closing speech:

Perhaps above all …we have [during this Conference] come to recognise …that there is a new and terrifying dimension of the smoking problem: …the spread of the smoking epidemic …to developing countries.^64^

Over the next fifteen years, there were a growing number of speeches, conferences papers, editorials, articles and reports by public health experts acknowledging, examining and discussing this new dimension of the smoking problem.^65^ Following Muller and Wickstrom, these experts argued that the spread of the smoking epidemic across the Third World was due to the tobacco industry’s ‘expansionary approach’: faced with ever more stringent anti-smoking policies and decreasing cigarette consumption in the West, the industry was hard at work establishing new markets for its products across Latin America, Asia and Africa through ‘highly sophisticated and ruthless [marketing] campaigns’.^66^ These experts also showed that, in developing countries, the smoking epidemic did not just lead to an increased chronic disease burden but also to food shortages and deforestation as farmers planted tobacco in lieu of edible crops and flue cured their harvest with firewood.^67^ All this, the experts feared, would ‘place an extra burden on developing countries already beset by problems of malnutrition and communicable disease’.^68^

The second development that helped shift the understanding of international action during the 1980s and early 1990s was the articulation of strategies to build tobacco control capacity in Third World countries. One strategy, that was first articulated by Lars Ramstrom at the 1979 Stockholm meeting and continued to be used at World Conferences throughout the second period, was to invite and fund doctors, health advocates and government officials from developing countries to attend an international convention to introduce them to and teach them about tobacco control.^69^ Another strategy, which was pioneered by Nigel Gray and his team at UICC before being later replicated on a much smaller scale by Roberto Masironi at WHO, was to fly North American and European experts to developing countries to run workshops with local physicians, community activists and public health officials to teach them about tobacco control.^70^ The organisation of such a workshop was a complex task.^71^ It started with a country visit to explore the place and identify local partners with the assistance of the local cancer society or the WHO country representative. The workshop itself was a combination of courses, group exercises and discussions run by a few foreign experts for a week. Its aim was not only to teach the participants about tobacco control but also to organise them into a national anti-smoking coalition and have them develop a comprehensive national tobacco control policy. To ensure that the workshop had wide exposure, it usually also involved an official speech by the country’s Ministry of Health or another dignitary as well as a broadcast about smoking on national television or radio with the foreign experts. Over time, these workshops often translated into the development of regional coordinating structures such as the *Comité Latino Americano Coordinador del Control del Tabaquismo*. Set up by the UICC representatives for South America, Allan Erickson and Carlos Herrera, the *Comité* allowed tobacco control leaders from Latin American countries to meet once a year to exchange information on their work, discuss the latest developments in the field and agree on a common strategy.^72^

As they had in the 1960s and 1970s, the World Conferences continued to provide, in conjunction with the UICC and WHO workshops, a space where the international tobacco control movement could develop and consolidate. In many ways, the movement in the 1980s and 1990s was a continuation of the networks and values developed in the earlier period, with many of the first generation of advocates such as Gray and Ramstrom still active during the second stage. But, there were also important ruptures. First, there was a shift in the professional profiles and nationalities of many of the individuals that joined the movement during the 1980s and early 1990s. While doctors and epidemiologists continued to be involved, many of the new members were economists like Kenneth Warner, lawyers like Ruth Roemer, sociologists like Simon Chapman, development specialists like David Nostbakken or professional campaigners like Mike Daube and Allan Erickson. So too, and this was a result of the problematisation of smoking in the Third World, the movement grew to include an ever-greater number of individuals from developing countries, such as Hong Kong physician Judith Mackay, South African public health specialist Derek Yach, Indian epidemiologist Prakash Gupta, Brazilian health expert Vera Luiza de Costa e Silva and Argentinian doctor Carlos Herrera. Second, there was also a shift in the movement’s collective identity and, specifically, in the way it construed its relationship with the tobacco industry. Up to the late 1970s, many anti-smoking activists genuinely felt that collaboration with the industry, as with the development of low-tar cigarettes, could be beneficial to the fight against smoking.^73^ The way representatives from cigarette companies openly attended the first World Conferences is perhaps symptomatic of this somewhat equivocal relationship with the industry during this early period.^74^ Attitudes quickly harden after that, with activists increasingly describing their relation with the industry as ‘a war’.^75^ The reasons for this shift were varied. Some public health activists thought that having an enemy would help unite the anti-smoking movement and the fight against tobacco.^76^ Others were wary of the industry’s persistent refusal to acknowledge the dangers of smoking.^77^

A large part of the knowledge developed and disseminated at the World Conferences, the WHO expert committees and the UICC workshops during the 1980s and early 1990s was action oriented, as it had been in the preceding period.^78^ While education and legislative action remained central,^79^ new types of strategies were developed. One of these was ‘clean indoor air policies’.^80^ The development of these policies was furthered by the pioneering research on passive smoking by Japanese epidemiologist Takeshi Hirayama, who gave one of the keynote speeches on his work at the 1987 World Conference in Tokyo.^81^ Another new strategy was advocacy tactics to monitor and counter the tobacco industry.^82^ The emergence of this strategy was a direct consequence of both the hardening attitudes towards the tobacco industry and the realisation that one had to tackle the vector of the epidemic to have a fighting chance of winning the war.^83^ But, what was really unique to the expertise produced during the 1980s and early 1990s was the articulation of an original body of knowledge on tobacco use in Third World societies.^84^ The need for such a body of knowledge came from the belief that ‘the smoking problem’ in developing countries ‘varied greatly’ from that in ‘industrialised nations’.^85^ As American tobacco control activist Karen Slama explained at the 1994 World Conference:

The economic and cultural factors vary widely throughout the world, and the fight against tobacco, which started in some countries over forty years ago is only beginning in some parts of the world.^86^

This meant that experts had to examine these varying economic and cultural conditions and adapt existing tobacco control strategies to make them work.^87^ A powerful example is the many reports and scholarly articles on smokeless forms of tobacco use such as the South Asian practice of betel chewing.^88^ This body of work, which explores the biochemistry, prevalence and health effects specific to these forms of tobacco use, led international experts not only to adapt their policies but also to change the name of their field from ‘smoking’ to ‘tobacco’ control to accommodate these different cultural practices.^89^

Overall, the funding structure for international action in the 1980s and early 1990s did not differ much from the one that had existed beforehand. Apart from the couple of positions at the WHO Tobacco or Health Programme, most advocates active in the world anti-smoking movement were employed by national organisations involved in tobacco control. Their international activities were carried out in addition to their day jobs and were generally unpaid. So, for example, the North American and European experts who organised the UICC workshops were all volunteers who would use their annual leave to fly to South America, Asia or Africa to teach about smoking control.^90^ National anti-smoking organisations and ACS, in particular, also remained the main source for funding the usual costs associated with international efforts.^91^ But, with the problematisation of smoking in the Third World, some of these costs also began to be shouldered by national development agencies. For example, while ACS paid for much of the transportation and accommodation costs incurred by the UICC experts when holding their workshops, it was the Swedish Development Agency that financed the WHO’s capacity building efforts in Asia and Africa.^92^ Similarly, it was funds from the same two institutions, supplemented with money from the Norwegian, Danish and Canadian development agencies, that enabled World Conference organisers to fly in delegates from poor developing countries.^93^

### 3.3 Stage 3: Globalisation and Tobacco, Early 1990s to Late 2000s

The post Cold War period between the early 1990s and the late 2000s saw a multiplication of international tobacco control efforts, with the WHO FCTC and the Bloomberg Initiative to Reduce Tobacco Use in Developing Countries (BI) being the most significant ones. The making of the FCTC was part of a broader drive at WHO at the time to seriously engage with and lead the international fight against chronic diseases.^94^ First articulated by American lawyers Ruth Roemer and Allyn Taylor in the early 1990s, the idea of the FCTC was only turned into reality after Gro Harlem Brundtland became WHO Director-General in 1999.^95^ Emboldened by the successful lawsuits against the tobacco industry in the USA and convinced by new epidemiological data that showed the rising global death toll related to smoking, in particular Oxford Professor Richard Peto’s global smoking-related mortality estimates and Chris Murray’s Global Burden of Disease calculations at Harvard, Brundtland quickly decided to make tobacco the prime focus of her WHO tenure, together with malaria. The FCTC was successfully adopted in 2003 after five years of often arduous negotiations led by the Tobacco Free Initiative (TFI) – a special, high-level WHO unit directed by Derek Yach and, later, Vera Luiza de Costa e Silva – with the support of a range of medical organisations, anti-smoking groups and consumer associations organised into the FCA. Besides laying out international rules on tobacco control, the FCTC also established two permanent, supranational institutions to ensure the application of these standards: the Permanent Secretariat and the Conference of the Parties.

The BI, which is still ongoing today, is an over US$ 500 million initiative to curb the tobacco epidemic in LMICs set up by businessman and then New York City mayor Michael Bloomberg in the mid-2000s with some support from the Bill and Melinda Gates Foundation (BMGF).^96^ Purposefully circumventing the FCTC institutions and national governments,^97^ the BI operates with a few, mostly American partners such as the TFI, the US Centers for Disease Control and Prevention (CDC), the CTFK and the UITLD. Working with local non-governmental organisations (NGOs) and some government agencies, which they fund through a small grants programme, the BI partners seek to implement their own selection of measures – the MPOWER package – that includes taxation, education and epidemiological surveillance. Besides the FCTC and the BI, there were also a few smaller international anti-smoking initiatives during this period. One, of course, was the World Conferences, which were held in Paris in 1994, in Beijing in 1997, in Chicago in 2000, in Helsinki in 2003, in Washington in 2006 and in Mumbai in 2009 and continued to provide an important meeting place for the anti-smoking movement.^98^ A similar, albeit virtual meeting place was established through GlobaLink, another small initiative led by ACS and UICC, which had, by then, terminated its Smoking and Lung Cancer Programme. Illustrative of technology’s critical role in the internationalisation of health, GlobaLink was a computer-based communication platform accessible through the worldwide web that enabled anti-smoking activists to receive daily news bulletins and exchange information in real time.^99^ There were also a number of efforts to build tobacco control expertise and research in the global South funded by North American development agencies and private philanthropies during this period.^100^ They included: efforts by the World Bank to develop tobacco taxation strategies in LMICs, which led to the publication of the influential report *Curbing the Epidemic*;^101^ the Rockefeller Foundation’s Trading Tobacco for Health Initiative to educate and organise advocates in South-East Asia;^102^ and both the Canadian IDRC’s Research on International Tobacco Control Initiative and the National Institutes of Health Fogarty International Centre’s International Tobacco and Health Research and Capacity Building Programme, which funded tobacco control research in Latin America, Asia and Africa.^103^

This third period was also marked by the reconfiguration of international action around the idea of globalisation, displacing earlier understandings centred on cross-national information sharing and development. From the 1990s onwards, a growing number of public health experts began talking about ‘global health’ rather than ‘international health’ or ‘health and development’.^104^ Drawing on the then pervasive theories on globalisation, these commentators believed that the world was undergoing a process of economic, political and social integration. For them, trade liberalisation and a revolution in communication and transportation technologies were leading to ever growing flows of information, goods, capital and people across political and geographical boundaries. Inherent to these processes of globalisation, they warned, were new types of threats to human health, from the rapid propagation of infections enabled by the development of air travel to the global spread of fatty foods, alcoholic beverages and cigarettes made possible by the abolition of trade barriers. For these commentators, the existing international health architecture centred on the nation-state was not equipped to deal with these new problems. Indeed, their transnational nature meant that these new types of threats were immune to the efforts of individual nation-states. This was further compounded by a profound scepticism towards the state, frequently regarded as wasteful and incompetent and fuelled by the then dominant neoliberal tradition.^105^ This led these commentators to develop and promote new, global forms of action that favoured working through non-state actors such as NGOs and philanthropists. These included: international regulatory frameworks; innovative financing mechanisms such as the Global Fund; transnational advocacy campaigns; private–public partnerships; and global epidemiological surveillance systems.

Unsurprisingly, these ideas about globalisation and new global forms of action could be found at work in most post Cold War, international tobacco control initiatives. The FCTC provides an excellent illustration. For Brundtland and her allies, an international treaty such as the FCTC was an innovative, global solution that made it possible to address the growing mortality associated with transnational cigarette marketing and trade which individual states could not tackle on their own. As she made clear:

The actions of individual countries can be ineffectual, primarily because of the globalisation of trade, marketing and information. Corporate interests in profits are no longer confined by geography …[and] national borders. [It is] in an effort to address the transnational aspects of tobacco control [that] the WHO has been working …[on] the FCTC.^106^

Another example is GlobaLink. The ACS-UICC’s internet-based platform was viewed by public health experts such as Derek Yach and his WHO colleague Douglas Bettcher as a ‘new global response’ to ‘the major threat to public health worldwide’ associated with the ‘globalisation of tobacco marketing, trade, research and industry influence’.^107^ Indeed, as one of these ‘new communications technologies’ developed at the time, they further argued, GlobaLink ‘profoundly improved’ the ‘prospects for effective global health advocacy’ by allowing activists around the world to interact simultaneously.^108^ The BI is a further example. Its preference to work through NGOs, both international and local, rather than the FCTC institutions and national governments reflects the neoliberal suspicion for the state and the United Nations’ bureaucracy that was dominant at the time. As one expert working for the BI explained:

We prefer to give money to an NGO …We think it is better than giving money to intergovernmental organisations like the WHO or to governmental or quasi-governmental organisations …It is because of the bureaucracy, the slowness in getting things done.^109^

Likewise, the way the experts administrating the BI have set up and use data generated by the Global Adult Tobacco Control Survey (GATS), a standardised survey run by the CDC that tracks smoking prevalence among adults and other key tobacco control indicators in countries around the world,^110^ is typical of how contemporary public health experts draw upon new forms of global epidemiological surveillance to manage global health efforts like the BI.^111^

From the mid-1990s to the late 2000s, the international tobacco control movement grew considerably. While many of the figures that became part of the movement in the preceding period, such as Derek Yach and Judith Mackay, continued to play a critical role, they were joined by a large number of new activists. One of the reasons for this growth in numbers was the TFI’s efforts to build a strong civil society movement to provide support for the FCTC during the negotiations, with Yach and his colleagues actively recruiting new activists and helping them organise into the FCA.^112^ These efforts were continued by the BI, which sought to ‘accelerate’ the making of a ‘worldwide movement to combat tobacco’, as well as other smaller capacity building projects like the Rockefeller’s initiative which led to the creation of the Southeast Asia Tobacco Control Alliance.^113^

Naturally, the movement’s growth was accompanied by changes in its membership and outlook. To start with, there was an increase in the number of advocates from the global South, with activists from Brazil, India, Thailand and many sub-Saharan African countries playing a critical role in the FCTC negotiations.^114^ There was also a rise in female participation, with active efforts made to attract women.^115^ Furthermore, there was also a growing proportion of professional campaigners and activists recruited from non-health backgrounds and, in particular, the environmental and consumer movements.^116^ More importantly perhaps was the extreme and previously unseen levels of loathing for the tobacco industry among activists, with most of them refusing to have any contact with an ‘enemy’ which they considered ‘evil’ and ‘absolutely vicious’.^117^ This radicalisation was the result of the access granted to the industry’s internal documents following the Master Settlement Agreement in the USA and the ‘sense of betrayal’ and ‘shock’ among activists when discovering the extent of the industry’s tactics.^118^

As had been the case previously, most of the knowledge produced during the third period was action oriented. This is hardly surprising given that the two most important initiatives at the time – the FCTC and the BI – were all about developing and implementing a set of effective strategies to tackle the smoking epidemic. Advertising bans, public education, smoke-free environments and industry monitoring remained central among the anti-smoking measures listed in both the FCTC and the MPOWER package.^119^ These familiar strategies were complemented in the post Cold War period with a series of new measures, including illicit trade policies, economic support for ex-tobacco farmers and, especially, taxation – which was now viewed as ‘the single most effective way to decrease tobacco use’.^120^ Identified as an anti-smoking measure in the 1970s already, taxation had remained unpopular and misunderstood among public health experts until the late 1980s.^121^ This only began to change when the extensive body of knowledge on tobacco taxes developed by American and European health economists in the 1970s and 1980s progressively found its way into the international tobacco control arena, first through papers at the World Conferences and then, decisively, through the work of the World Bank.^122^

Another significant development in the post Cold War period was the production of a new body of epidemiological data on global smoking prevalence and smoking-related mortality and morbidity. This was part of broader shift towards evidence-based approaches to policy making in global health at the time.^123^ There had been some efforts by the UICC and WHO to set up and standardise national epidemiological surveillance systems in the 1980s.^124^ But, up to the 1990s and often beyond, most developing countries did not collect data on smoking habits and smoking-related mortality, and the global estimates that did exist, such as those from the WHO, were ‘always questioned’ and deemed to ‘simply be wrong’.^125^ The work on global, smoking-attributable mortality carried out by Richard Peto and his colleagues were among the first attempts to address this lack of reliable data. Combining sophisticated modelling techniques, population and mortality statistics collected by the WHO and large observational studies, this work made clear, for the first time, that tobacco was the leading global cause of death and played a critical role in the adoption of the FCTC.^126^ Peto’s work was followed by other attempts to collect and calculate global epidemiological data such as the ACS’ Nations database and, more recently, the CDC’s GATS project.^127^

Finally, it is important to stress the shift in terms of the funding structure that took place at the end of the 1990s in the global field of tobacco control. Of course, funds from national anti-smoking organisations and development agencies did not dry up. So, for example, ACS continued to be generous, financing among others the World Conferences and GlobaLink, and the Norwegian Cancer Society helped fund the work of the FCA.^128^ Similarly, the Canadian IDRC, as part of its Research on International Tobacco Control Initiative, was an important source of funding for research into tobacco control in the developing world.^129^ But all these contributions were dwarfed by the important sums of money now poured in by American philanthropists, echoing the historical involvement of philanthropic organisations, such as Rockefeller, in international health.^130^ The first one to do so was the United Nations Foundation set up by American media mogul Ted Turner, which paid for activists from developing countries to attend the FCTC negotiations.^131^ It was followed by the efforts of Rockefeller, Gates and Bloomberg, already mentioned above. The money invested by these philanthropists led to the ‘professionalisation’ of international tobacco control in the global North, where the WHO headquarters and many American NGOs such as ACS and CTFK would now hire full-time staff or pay for expensive consultants to do their international work.^132^ In the global South, in contrast, much of the work in this field has remained precarious or voluntary.

## 4 Conclusion

This article explored the history of the internationalisation of tobacco control. In the existing literature, this history is often reduced to the making of the FCTC. If other international efforts are treated at all, they are typically read as steps that necessarily lead to the adoption of the WHO treaty at the start of the twenty-first century. Avoiding these reductive and Whiggish narratives, this article sought to paint a more complex and contrasted picture of the history of international tobacco control. To start with, it showed that this history is much longer and richer than the making of the FCTC. Indeed, the internationalisation of anti-smoking efforts started in the 1950s through informal contacts between individual scientists working on cancer research. These initial interactions were followed, from the mid-1960s onwards, by a growing number of more formal initiatives. The World Conferences and the UICC Smoking and Lung Cancer Programme were the most prominent among these up to the late 1980s. More recently, they have been eclipsed by the launch of the internet-based communication platform GlobaLink, the making of the FCTC and the implementation of the BI.

Echoing anthropologists’ claims about global health’s fractured terrain, the article also emphasised the ruptures and discontinuities that mark the history of international tobacco control. Specifically, we identified three successive periods, all characterised by different notions of international action, tobacco control expertise, advocacy networks and funding strategies. We showed how international action, which, at first, was primarily understood as experts from individual countries coming together to share ideas and support each other’s national efforts, became progressively viewed through a development lens during the late 1970s and 1980s before being re-articulated around the idea of globalisation at the end of the twentieth century. Similarly, we revealed how the networks of advocates and experts that made up the world movement against smoking changed considerably over time. Specifically, we showed how what started as a small, hardly ‘global’ group of male physicians from North America and Europe gradually grew in size and opened up to women, representatives from the developing world and other occupations such as lawyers, economists and professional activists. We also showed how this movement’s relationship with the tobacco industry shifted radically, from collaborations to develop less harmful cigarettes to talk of war and full rejection. Furthermore, we also demonstrated how both the object of action and preferred type of intervention in international tobacco control changed over time with the articulation of new bodies of expertise, from the research on other cultural forms of tobacco use to health economists’ work on taxation.

Placed within the longer history of international health regulation, some aspects of this history of tobacco control very much resonate with the past. The transnational networks of doctors, activists and lawyers so critical to the internationalisation of tobacco control are akin to the networks of experts who operated through the League of Nations in the inter-war years.^133^ Likewise, the World Conferences draw on a form of transnational collaboration that has been present since the nineteen-century’s international health conferences on cholera.^134^ Furthermore, the key role played by national organisations and, later on, private philanthropists, especially US-based ones, in providing the necessary resources for international tobacco control efforts echoes the part played by private American philanthropy in the inter-war period.^135^ But, more importantly, perhaps, and as other scholars have argued,^136^ the history of international tobacco control presented in this article shows the value of looking at the past when trying to make making sense of contemporary global health landscapes.

## Footnotes

1.
As historians and other scholars have shown, there was a reconfiguration of how world health was conceptualised at the end of the Cold War (N. King, ‘Security, Disease, Commerce’, *Social Studies of Science*, 32 (2002), 763–89; T.M. Brown, M. Cueto and E. Fee, ‘The World Health Organization and the Transition from “International” to “Global” Public Health’, *American Journal of Public Health*, 96 (2006), 62–72; D. Fassin, ‘The obscure object of global health’, in M. Inhorn, E. Wentzell (eds), *Medical Anthropology at the Intersections* (Durham, NC: Duke University Press, 2012), 95–115; J.-P. Gaudillière, ‘De la santé publique internationale a la santé globale’, in D. Pestre (ed.), *Le Governement des technosciences* (Paris: Editions La Découverte, 2014), 65–96; D. Reubi, ‘Modernisation, Smoking and Chronic Disease’, *Health & Place*, 39 (2016), 188–195). This was marked by a terminological shift whereby ‘global’ progressively displaced ‘international’ as the term used to characterise transnational health-related initiatives without eliminating it altogether. As we show in this article, a similar shift, both conceptual and terminological, took place in tobacco control at around the same time. To mark this shift, we have used the word ‘international’ (and variants thereof like ‘transnational’) to describe cross-national initiatives that took place at any point in time and have limited our use of the word ‘global’ to talk about transnational efforts that happened from the early 1990s onwards.

2.
See, for example: J. Collin, K. Lee and K. Bissell, ‘The Framework Convention on Tobacco Control: the Politics of Global Health Governance’, *Third World Quarterly*, 23 (2002), 265–82; R. Roemer, A. Taylor and J. Larivière, ‘Origins of the WHO Framework Convention on Tobacco Control’, *American Journal of Public Health*, 95 (2005), 936–8; A. Brandt, *The Cigarette Century*: *The Rise, Fall and Deadly Persistence of the Product that Defined America* (New York: Basic Books, 2007), ch. 13; H.M. Mamudu, M.E. Gonzalez and S. Glantz, ‘The Nature, Scope and Development of the Global Tobacco Control Epistemic Community’, *American Journal of Public Health*, 101 (2011), 2033–54; P. Cairney, D.T. Studlar and H.M. Mamudu, *Global Tobacco Control: Power, Policy, Governance and Transfer* (Basingstoke: Palgrave MacMillan, 2012); L.A. Reynolds and E.M. Tansey (eds), *WHO Framework Convention on Tobacco Control: Witness Seminar Transcript* (London: Queen Mary University of London, 2012); D. Yach, ‘The Origins, Development, Effects and Future of the WHO Framework Convention on Tobacco Control’, *The Lancet*, 383, 9930 (2014), 1771–9.10.1016/S0140-6736(13)62155-824461287

3.
Roemer *et al.*, *op. cit.* (note 2).

4.
H.M. Mamudu and S.A. Glantz, ‘Civil Society and the Negotiation of the Framework Convention on Tobacco Control’, *Global Public Health*, 4 (2009), 150–68; WHO, *History of the WHO Framework Convention on Tobacco Control* (Geneva: WHO, 2009).10.1080/17441690802095355PMC266451819333806

5.
V. Berridge, ‘Introduction’, in L.A. Reynolds and E.M. Tansey (eds), *WHO Framework Convention on Tobacco Control: Witness Seminar Transcript* (London: Queen Mary University of London, 2012), xxi–xxvi.

6.
H. Butterfield, *The Whiggish Interpretation of History* (New York: Norton, 1965).

7.
See, for example: Brown *et al.*, *op. cit.* (note 1); A. Bashford (ed.), *Medicine at the Border: Disease, Globalisation and Security, 1850 to the Present* (Basingstoke: Palgrave MacMillan, 2007); S. Bhattacharya, ‘The World Health Organization and Global Smallpox Eradication’, *Journal of Epidemiology and Community Health*, 62 (2008), 909–12; S. Bhattacharya and R. Dasgupta, ‘A Tale of Two Global Health Programmes’, *American Journal of Public Health*, 99, 7 (2009), 1176–84; V. Berridge, K. Loughlin and R. Herring, ‘Historical dimensions of global health governance’, in K. Buse, W. Hein and N. Drager (eds), *Making Sense of Global Health Governance* (Basingstoke: Palgrave Macmillan, 2009), 28–46; Gaudillière, *op. cit.* (note 1).

8.
The domination of the World Conferences has changed over time. From 1967 to 1987, they were called ‘World Conference on Smoking and Health’. From 1990 onwards, in recognition that smoking was only one possible tobacco use that was dangerous for human health, ‘smoking’ was replaced by ‘tobacco’. From 1992 onwards, in recognition that smoking/tobacco and health were mutually exclusive, the ‘and’ was replaced by ‘or’. Thus, from then on, the World Conferences were called ‘World Conferences on Tobacco or Health’.

9.
See, for example: King, *op. cit.* (note 1); Fassin, *op. cit.* (note 1); W. Anderson, ‘Making Global Health History’, *Social History of Medicine*, 27 (2014), 372–84; Reubi, *op. cit.* (note 1).

10.
For obvious language proficiency reasons, the collection was limited to articles written in English, French, German and Spanish.

11.
Reynolds and Tansey, *op. cit.* (note 2).

12.
Brandt, *op. cit.* (note 2); V. Berridge, *Marketing Health: Smoking and the Discourse of Public Health in Britain, 1945–2000* (Oxford: Oxford University Press, 2007).

13.
As Robert Proctor has showed (*The Nazi War on Cancer*, Princeton, NJ: Princeton University Press, 1999), there were earlier anti-tobacco control efforts but their international character was very limited. One such example is Argentinian physician Angel Roffo who helped establish that tobacco tar was carcinogenic in the 1920s and published much of his work in German cancer journals. Another example is the physicians working in Nazi Germany in the late 1930s and early 1940s, who were among the first to establish an epidemiological link between smoking and lung cancer, more than a decade before their British and American counterparts. Probably because of the disruptions caused by the war, these physicians did not really establish links with scientists in other countries, apart from a few collaborations with doctors in Nazi occupied Czechoslovakia.

14.
Berridge, *op. cit.* (note 12).

15.
N.C. Delarue, ‘Smoking or health activities’, in W.F. Forbes, R.C. Frecker and D. Nostbakken (eds), *Proceedings of the 5th**World Conference on Smoking and Health* (Ottawa, Canadian Council on Smoking and Health, 1985), 797.

16.
Berridge, *op. cit.* (note 12); Brandt, *op. cit.* (note 2).

17.
D. Armstrong, ‘The Rise of Surveillance Medicine’, *Sociology of Health & Illness*, 17 (1995), 393–404; A. Petersen and D. Lupton, *The New Public Health: Discourses, Knowledges, Strategies* (London: Sage, 2000); G. Weisz, *Chronic Disease in the Twentieth Century: a History* (Baltimore, MD: Johns Hopkins University Press, 2014); D. Armstrong, ‘Chronic Disease: a Revisionist Account’, *Sociology of Health & Illness*, 36 (2014), 15–27.

18.
H.A. Goodman (ed.), *World Conference on Smoking and Health* (New York: American Cancer Society, 1968); R.G. Richardson (ed.), *The Second World Conference on Smoking and Health* (London: Pitman Medical, 1971); J. Steinfeld **et al.** (eds), *Proceedings of the 3rd* *World Conference on Smoking and Health* (Bethesda, MD: National Cancer Institute, 1977).

19.
G. Godber, ‘It can be done’, in R.G. Richardson (ed.), *The Second World Conference on Smoking and Health* (London: Pitman Medical, 1971), 141–7; G. Godber, ‘Presentation to the Fifth World Conference on Smoking and Health’, in W.F. Forbes, R.C. Frecker and D. Nostbakken (eds), *Proceedings of the 5th* *World Conference on Smoking and Health* (Ottawa: Canadian Council on Smoking and Health, 1985), 71–5; R. Roemer, *Legislative Action to Combat the World Tobacco Epidemic*, 2nd edn (Geneva: WHO, 1993), 1–6.

20.
WHO, *Smoking and its Effects on Health* (Geneva: WHO, 1975); WHO, *Controlling the Smoking Epidemic* (Geneva: WHO, 1979); Roemer, *op. cit.* (note 19), annex II.

21.
Godber, *op. cit.* (note 19), 142.

22.
N. Gray, ‘Smoking and Lung Cancer’, *World Smoking & Health*, 1 (1976), 35–6; N. Gray (ed.), *Guidelines for Smoking Control*, 1st edn (Geneva: UICC, 1977).

23.
Godber, *op. cit.* (note 19), 146.

24.
P. Weindling (ed.), *International Health Organisations and Movements* (Cambridge: Cambridge University Press, 1985); Bashford, *op. cit.* (note 7); A.E. Birn, ‘The Stages of International (Global) Health: Histories of Success or Successes of History?’, *Global Public Health*, 4, 1 (2009), 50–68.10.1080/1744169080201779719153930

25.
Goodman, *op. cit.* (note 18); Richardson, *op. cit.* (note 17); Steinfeld *et al.*, *op. cit.* (note 18).

26.
Goodman, *op. cit.* (note 18), i.

27.
Goodman, *op. cit.* (note 18), 2.

28.
W.S. Ross, ‘Editorial’, *World Smoking & Health*, 1 (1976), 2.

29.
Steinfeld *et al.*, *op. cit.* (note 18), xvi.

30.
K. Farquharson ‘Influencing Policy Transnationally’, *Australian Journal of Public Administration*, 4, 2 (2003), 80–92; Mamudu *et al.*, *op. cit.* (note 2); D. Reubi, ‘Constructing tobacco control as a human rights issue’, in A. Mold and D. Reubi (eds), *Assembling Health Rights in Global Context: Genealogies and Anthropologies* (Oxon: Routledge, 2013), 109–26. More generally, see: P. Hass, ‘Epistemic Communities and International Policy Coordination’, *International Organization*, 46, 1 (1992), 1–35; M.E. Keck and K. Sikkink, *Activists Beyond Borders* (Ithaca, NY: Cornell University Press, 1998); S. Epstein, ‘Patient groups and health movements’, in E.J. Hackett **et al.** (eds), *The Handbook of Science and Technology Studies*, 3rd edn (Cambridge, MA: MIT Press, 2008), 499–540.

31.
Interview, May 2014.

32.
N. Gray and M. Daube (eds), *Guidelines for Smoking Control*, 2nd edn (Geneva: UICC, 1980); A. Lopez, N. Collishaw and T. Piha, ‘A Descriptive Model of the Cigarette Epidemic in Developed Countries’, *Tobacco Control*, 3 (1994), 242–7.

33.
Quoted in Goodman, *op. cit.* (note 18), 1.

34.
L. Ramstrom, ‘The 4th World Conference on Smoking and Health’, *World Smoking & Health*, 3 (1978), 50.

35.
Goodman, *op. cit.* (note 18), v–x; Richardson, *op. cit.* (note 18), vii–x; Steinfeld *et al.*, *op. cit.* (note 18), xv–xxvi.

36.
WHO, *Smoking**op. cit.* (note 20); R. Masironi, ‘Controlling the Smoking Epidemic’, *World Smoking & Health*, 4 (1979), 4–8; WHO, *Controlling* *op. cit.* (note 20), 3–4.

37.
Interview, September 2013. See also Gray, *Smoking*, *op. cit.* (note 22), 35.

38.
Goodman, *op. cit.* (note 18), 231. See, for example: Steinfeld *et al.*, *op. cit.* (note 18), 231–454; Gray, *Smoking*, *op. cit.* (note 22); 13–52; WHO, *Controlling*, *op. cit.* (note 20), 45–52.

39.
See, for example: Goodman, *op. cit.* (note 18), 238–49; Gray, *Smoking*, *op. cit.* (note 18), 64–75; WHO, *Controlling*, *op. cit.* (note 20), 55–7.

40.
Richardson, *op. cit.* (note 18), vii. See, for example: C. Fletcher, ‘The lines to pursue’, in R.G. Richardson (ed.), *The Second World Conference on Smoking and Health* (London: Pitman Medical, 1971), 157–62; Gray, *Guidelines*, *op. cit.* (note 22), 54–9; F. Schmidt, ‘Defused Cigarettes – Slogan or Reality?’, *World Smoking & Health*, 2 (1978), 35–9.

41.
Goodman, *op. cit.* (note 18), 10. See, for example: Steinfeld *et al.*, *op. cit.* (note 18), 455–532; WHO, *Controlling*, *op. cit.* (note 18), 66–71; Gray, *Guidelines*, *op. cit.* (note 22), 52.

42.
Keck and Sikkink, *op. cit.* (note 30), 12.

43.
Interview, June 2014.

44.
Goodman, *op. cit.* (note 18); Fletcher, *op. cit.* (note 40); Steinfeld *et al.*, *op. cit.* (note 18); Gray, *Guidelines*, *op. cit.* (note 22); M. Daube, ‘Summing up of the IV World Conference on Smoking and Health’, in L.M. Ramstrom (ed.), *The Smoking Epidemic* (Stockholm: Almqvist and Wiksell, 1979), 322–37.

45.
Interview, January 2014.

46.
L.M. Ramstrom (ed.), *The Smoking Epidemic* (Stockholm: Almqvist and Wiksell, 1979); W.F. Forbes, R.C. Frecker and D. Nostbakken (eds), *Proceedings of the 5th* *World Conference on Smoking and Health* (Ottawa: Canadian Council on Smoking and Health, 1985); M. Aoki, S. Hisamachi and S. Tominaga (eds), *Smoking and Health 1987.* (Amsterdam: Elsevier, 1988); B. Durston and K. Jamrozik (eds), *Tobacco and Health, 1990* (Perth: Health Department of Western Australia, 1990); American Cancer Society, *Eighth World Conference on Tobacco or Health* (Atlanta, GA: American Cancer Society, 1994).

47.
M. Peng, ‘The urgent need to control the smoking epidemic in the Third World’, in W.F. Forbes, R.C. Frecker and D. Nostbakken (eds), *Proceedings of the 5th**World Conference on Smoking and Health* (Ottawa: Canadian Council on Smoking and Health, 1985), 561–6.

48.
Daube, *op. cit.* (note 44); Interview, March 2012.

49.
Daube, *op. cit.* (note 44), 325–6; H. Mahler, ‘Smoking or Health, the Choice is Yours’, *World Smoking & Health*, 5 (1980), WHO, *Global Strategy for Health for All by the Year 2000* (Geneva: WHO, 1981); H. Mahler, ‘Tobacco or health: choose health’, in M. Aoki, S. Hisamichi and S. Tominaga (eds), *Smoking and Health 1987* (Amsterdam: Elsevier, 1988), 9–14. On the primary health care agenda, see M. Cueto, ‘The Origins of Primary Health Care and Selective Primary Health Care’, *American Journal of Public Health*, 94 (2004), 1864–74.

50.
The programme was initially called the Smoking and Health Programme before changing its name to the Tobacco or Health Programme in 1988. On the programme, see: R. Masironi, ‘The Role of the WHO in smoking control’, in W.F. Forbes, R.C. Frecker and D. Nostbakken (eds), *Proceedings of the 5th**World Conference on Smoking and Health* (Ottawa: Canadian Council on Smoking and Health, 1985), 99–102; L. Ramstrom, ‘Smoking control in developing countries’, in W.F. Forbes, R.C. Frecker and D. Nostbakken (eds), *Proceedings of the 5th* *World Conference on Smoking and Health* (Ottawa: Canadian Council on Smoking and Health, 1985), 581–7; C. Chollat-Traquet, ‘WHO tobacco or health programme’, in B. Durston and K. Jamrozik (eds), *Tobacco and Health 1990* (Perth: Health Department of Western Australia, 1990), 314–6; C. Chollat-Traquet, ‘Tobacco or Health: a WHO Programme’, *European Journal of Cancer*, 28 (1992), 311–5.

51.
Berridge, *op. cit.* (note 5), xxiii; Reynolds and Tansey, *op. cit.* (note 2), 15 and 48.

52.
WHO, *Smoking Control Strategies in Developing Countries*, (Geneva: WHO, 1983); WHO, *Smokeless Tobacco Control* (Geneva: WHO, 1988).

53.
See, for example: R. Roemer, *Legislative Action to Combat the World Smoking Epidemic*, 1st edn (Geneva: WHO, 1982); C. Chollat-Traquet, *Women and Tobacco,* (Geneva: WHO, 1992).

54.
Masironi, *op. cit.* (note 50); Ramstrom, *op. cit.* (note 50); Chollat-Traquet, *WHO*, *op. cit.* (note 50).

55.
Pan-American Health Organization, *Smoking Control: Third Sub-Regional Workshop, Caribbean Area, Kingston Jamaica, 8–11 December 1987* (Washington DC: Pan-American Health Organization, 1988); WHO Europe, *A Five-Year Action Plan* (Copenhagen: WHO Europe, 1988); H. Chitanondh, ‘The role of regional organisations in the Asia Pacific Region’, in K. Slama (ed.), *Tobacco and Health* (New York: Plenum Press, 1994), 255–60; Reynolds and Tansey, *op. cit.* (note 2), 9 & 16.

56.
Daube, *op. cit.* (note 44), 324; Chollat-Traquet, *WHO*, *op. cit.* (note 50), 315. On the programme, see: A. Erickson, ‘Beyond UICC sponsored workshops’, in B. Durston and K. Jamrozik (eds), *Tobacco and Health, 1990* (Perth: Health Department of Western Australia, 1990), 130–1; D. Nostbakken, ‘UICC smoking control activities in Africa’, in B. Durston and K. Jamrozik (eds), *Tobacco and Health, 1990* (Perth: Health Department of Western Australia, 1990), 169–78; M. Wood, ‘Overview and purpose of national/regional strategic planning sessions from Buenos Aires to Paris’, in American Cancer Society, *Eighth World Conference on Tobacco or Health* (Atlanta, GA: American Cancer Society, 1994), 84–9.

57.
See, for example: Gray and Daube, *op. cit.* (note 32); M. Pertschuk, *Smoke Signals* (Geneva: UICC, 1987).

58.
A product of the Cold War, decolonisation and development, the notion of ‘Third World’ was coined by French demographer Alfred Sauvey in the early 1950s. While considered problematic today, it was a widely accepted term commonly used to refer to the developing world until the early 1990s (A. Escobar, *Encountering Development*, Princeton, NJ: Princeton University Press, 1995; M. Mazower, *Governing the World*, London: Penguin Books, 2012).

59.
See, for example: J. Bryant, *Health and the Developing World* (Ithaca, NY: Cornell University Press, 1969); A.R. Omran, ‘The Epidemiological Transition’, *Milbank Memorial Fund Quarterly*, 49 (1971), 509–38; F. Brockington, *The Health of the Developing World* (Lewes: Book Guild, 1985).

60.
D. Reubi, ‘Health Economists, Tobacco Taxes and International Development’, *BioSocieties*, 8 (2013), 205–28; Weisz, *op. cit.* (note 17); D. Reubi, C. Herrick, T. Brown, ‘The Politics of Non-Communicable Diseases in the Global South’, *Health & Place*, 39 (2016), 179–187.10.1016/j.healthplace.2015.09.001PMC488978626365886

61.
M. Muller, *Tobacco and the Third World* (London: War on Want, 1978); B. Wickstrom, *Cigarette Marketing and the Third World* (Gothenburg: University of Gothenburg, 1979).

62.
B. Wickstrom, ‘Cigarette marketing in the Third World’, in L.M. Ramstrom (ed.), *The Smoking Epidemic* (Stockholm: Almqvist and Wiksell, 1979), 98–105.

63.
Interview, June 2014.

64.
Daube, *op. cit.* (note 44), 323.

65.
See, for example: WHO, *Developing*, *op. cit.* (note 52); J. Crofton, ‘The Gathering Smoke of Clouds’, *International Journal of Epidemiology*, 13 (1984), 269–70; Ramstrom, *op. cit.* (note 49); Mahler, *Tobacco*, *op. cit.* (note 49); S. Chapman and W. Wai Leng, *Tobacco Control in the Third World* (Penang: International Organisation of Consumers Unions, 1990); K.R. Stebbins, ‘Transnational Tobacco Companies and Health in Underdeveloped Countries’, *Social Science and Medicine*, 30 (1990), 227–35; J. Mackay, ‘Tobacco: the Third World War’, *Thorax*, 46 (1991), 153–6.

66.
WHO, *Developing*, *op. cit.* (note 52), 8–9.

67.
J. Madeley, ‘The environmental impact of tobacco production in developing countries’, in W.F. Forbes, R.C. Frecker and D. Nostbakken (eds), *Proceedings of the 5th**World Conference on Smoking and Health* (Ottawa: Canadian Council on Smoking and Health, 1985), 287–90; S. Chapman **et al.**, ‘Smoking Control in Africa’, *Health Policy & Planning*, 1 (1986), 222–31; Chapman and Wei Leng, *op. cit*. (note 65).

68.
Mahler, *Tobacco*, *op. cit.* (note 49), 12.

69.
Daube, *op. cit.* (note 44); Ramstrom, *op. cit.* (note 50).

70.
Gray and Daube, *op. cit.* (note 32); Ramstrom, *op. cit.* (note 50); Erickson, *op. cit.* (note 56); Nostbakken, *op. cit.* (note 56); Wood, *op. cit.* (note 56).

71.
See, for example: D. Simpson, *Report on Site Visit on Smoking and Cancer on the Indian Subcontinent* (Geneva: UICC, 1981); UICC, *Seminario Taller del Habito de Fumar y la Salud, Panama* (Geneva: UICC, 1984); WHO, *Seminaire-atelier regional de l’OMS pour les pays francophones d’Afrique, Lome, Togo, 25–30 novembre 1985* (Geneva: WHO, 1986).

72.
C.A. Herrera, ‘Fifth Annual Meeting of the Latin American Coordinating Committee on Smoking Control’, *World Smoking & Health*, 14 (1989), 13–14; Erickson, *op. cit.* (note 56).

73.
Goodman, *op. cit.* (note 18), 1–13; L. Kaprio, ‘Strengthening our resources by international collaboration’, in L.M. Ramstrom (ed.), *The Smoking Epidemic* (Stockholm, Almqvist and Wiksell, 1979), 298–301; S. Chapman, *The Lung Goodbye* (Penang: Consumer Interpol, 1983), 1–8. See also: Berridge, *op. cit.* (note 12), ch. 3.

74.
Daube, *op. cit.* (note 44), 328–9; M. Daube, ‘Public information programmes’, in W.F. Forbes, R.C. Frecker and D. Nostbakken (eds), *Proceedings of the 5th* *World Conference on Smoking and Health* (Ottawa: Canadian Council on Smoking and Health, 1985), 47–58: 47–8.

75.
See, for example: Daube, *op. cit.* (note 44), 48; Daube, *op. cit.* (note 73), 48; M. Pertschuk, ‘Waging War Against Tabaquismo in Latin America’, *World Smoking & Health*, 10 (1985), 21–2; S. Glantz, ‘Non-Smokers’ Rights Campaign: Guerilla Warfare in the Third World’, *World Smoking & Health*, 13 (1988), 26–8; W. Tipping, ‘The Next Area of the Global Tobacco War’, *World Smoking & Health*, 14 (1989), 2; Mackay, *op. cit.* (note 65), 153.

76.
Interview, March 2012; interview, July 2013.

77.
L. Terry, ‘Getting Away with Murder’, *World Smoking & Health*, 7 (1983), 2–3.

78.
Daube, *op. cit.* (note 44), 322; Durston and Jamrozik, *op. cit.* (note 46), v.

79.
See, for example: Gray and Daube, *op. cit.* (note 32), chs 6, 7 and 9; Roemer, *op. cit.* (note 53); WHO, *Developing*, *op. cit.* (note 52), sections 3.4 and 3.5; Daube, *op. cit.* (note 74); E. Gritz, ‘The challenge to public education’, in W.F. Forbes, R.C. Frecker and D. Nostbakken (eds), *Proceedings of the 5th* *World Conference on Smoking and Health* (Ottawa: Canadian Council on Smoking and Health, 1985), 301–10; J.F. Banzhaf, ‘Smoking control legislation’, in M. Aoki, S. Hisamachi and S. Tominaga (eds), *Smoking and Health 1987* (Amsterdam: Elsevier, 1988), 399–404.

80.
See, for example: J.L. Repace and A.H. Lowrey, ‘Indoor Air Pollution, Tobacco Smoke and Public Health, *World Smoking & Health*, 7 (1982), 4–13; J.E. Fielding, ‘Banning Worksite Smoking’, *World Smoking & Health*, 12 (1987), 3–4; L. L. Fairbanks, ‘Total smoking bans’, in B. Durston and K. Jamrozik (eds), *Tobacco and Health, 1990* (Perth: Health Department of Western Australia, 1990), 479–81; M.P. Eriksen, ‘Clean indoor air policies’, in American Cancer Society, *Eighth World Conference on Tobacco or Health* (Atlanta, GA: American Cancer Society, 1994), 71–8.

81.
T. Hariyama, ‘Non-Smoking Wives of Heavy Smokers Have a Higher Risk of Lung Cancer’, *British Medical Journal,*282 (1981), 183–5; T. Hirayama, ‘Health effects of active and passive smoking’, in M. Aoki, S. Hisamachi and S. Tominaga (eds), *Smoking and Health 1987* (Amsterdam: Elsevier, 1988), 75–86.

82.
See, for example: Chapman, *op. cit.* (note 73); Durston and Jamrozik, *op. cit.* (note 46), Section 8 on Activism and Industry Tactics; W. Godshall, ‘Exposing the Tobacco Lobby’, *World Smoking & Health*, 18 (1993), 10–11; L. Pinillos, ‘Countering tobacco marketing, advertising and promotion’, in American Cancer Society, *Eighth World Conference on Tobacco or Health* (Atlanta, GA: American Cancer Society, 1994), 34–40.

83.
Chapman, *op. cit.* (note 73), 1–8.

84.
See, for example: A. Taha and K. Ball, ‘Smoking in Africa’, *World Smoking & Health*, 7 (1982), 25–30; WHO, *Developing*, *op. cit.* (note 51); F. Lokschin and F. Barros, ‘Smoking or Health: the Brazilian Option’, *New York State Journal of Medicine*, 83 (1983), 1314–6; P.C. Gupta, ‘Health Consequences of Tobacco Use in India’, *World Smoking & Health*, 13 (1988), 5–10; X.Z. Weng, ‘Smoking: a serious problem in China’, in M. Aoki, S. Hisamachi and S. Tominaga (eds), *Smoking and Health 1987* (Amsterdam: Elsevier, 1988), 611–4.

85.
Gray and Daube, *op. cit.* (note 32), 6.

86.
K. Slama (ed.), *Tobacco and Health* (New York: Plenum, 1994), vii.

87.
Slama, *op. cit.* (note 86), v; Reynolds and Tansey, *op. cit.* (note 2); 25.

88.
See, for example: F.S. Mehta, P.C. Gupta and J.J. Pindborg, ‘Chewing and Smoking Habits in Relation to Precancer and Oral Cancer’, *Journal of Cancer Research and Clinical Oncology*, 99 (1981), 35–9; A.J. McMichael, ‘Oral Cancer in the Third World’, *International Journal of Epidemiology*, 13 (1983), 403–5; M.B. Aghi, P.C. Gupta and F. Mehta, ‘Intervention in the Tobacco Habits of Rural Indian Women’, *World Smoking & Health*, 9 (1984), 10–14; WHO, *Smokeless*, *op. cit.* (note 52).10.1007/BF00412440PMC122532927251636

89.
Mahler, *Tobacco*, *op. cit.* (note 49).

90.
Interview, June 2014.

91.
Interview, April 2013; interview, June 2014.

92.
Gray and Daube, *op. cit.* (note 32); Ramstrom, *op. cit.* (note 50).

93.
Ramstrom, *op. cit.* (note 46); Forbes, Frecker and Nostbakken, *op. cit.* (note 46).

94.
Weisz, *op. cit.* (note 17); Reubi, Herrick and Brown, *op. cit.* (note 60).

95.
On the FCTC, see: Roemer *et al.*, *op. cit.* (note 2); WHO, *op. cit.* (note 4); Mamadu and Glantz, *op. cit.* (note 4); Reynolds and Tansey, *op. cit.* (note 2); Yach, *op. cit.* (note 2).

96.
On the BI, see: T.R. Frieden and M. R. Bloomberg, ‘How to Prevent 100 Million Deaths from Tobacco’, *The Lancet*, 369, 9574 (2007), 1758–61; WHO, *WHO Report on the Global Tobacco Epidemic, 2009* (Geneva: WHO, 2009); Bloomberg Philanthropies, *Accelerating the Worldwide Movement to Reduce Tobacco Use* (New York: Bloomberg Philanthropies, 2011).10.1016/S0140-6736(07)60782-X17512860

97.
Reynolds and Tansey, *op. cit.* (note 2), 66.

98.
On the conferences, see: Slama, *op. cit.* (note 86); R. Lu *et al.* (eds), *Tobacco, the Growing Epidemic* (London: Springer, 2000).

99.
R. Israel, ‘GlobaLink’, in R. Lu *et al.* (eds.), *Tobacco, the Growing Epidemic* (London: Springer, 2000), 856–7.

100.
H.A. Lando *et al.*, ‘The Landscape in Global Tobacco Control Research’, *American Journal of Public Health*, 95 (2005), 939–45.10.2105/AJPH.2004.047167PMC144928815914813

101.
World Bank, *Curbing the Epidemic* (Washington, DC: World Bank, 1999). As Reubi *op. cit.* (note 60) has showed, the publication of *Curbing the Epidemic* represented a radical shift in the way the Bank viewed tobacco. Indeed, until then, the Bank considered the plantation of tobacco crops and the manufacture of cigarettes as purely beneficial activities that led to economic growth and development and which it therefore actively supported through grants.

102.
Rockefeller Foundation, *Annual Report, 2002* (New York: Rockefeller Foundation, 2002).

103.
IDRC, ‘Research on International Tobacco Control’, available at http://web. idrc. ca (consulted 12th July 2012); Fogarty International Centre, ‘Awards for the International Tobacco and Health Research and Capacity Building Program’, available at http://www.fic.nih.gov/Grants/Search/Pages/Awards-Program-Tobacco.aspx (consulted 5 April 2012).

104.
King, *op. cit.* (note 1); Brown, Cueto and Fee, *op. cit.* (note 1); Fassin, *op. cit.* (note 1); Gaudilliere, *op. cit.* (note 1); Reubi, *op. cit.* (note 1). See, for example: G. Walt, ‘The Globalisation of International Health’, *The Lancet*, 351 (1998), 434–7; D. Yach and D. Bettcher, ‘The Globalisation of Public Health’, *American Journal of Public Health*, 88 (1998), 735–41; N. Daulaire, ‘Globalization and Health’, *Development*, 42, 4 (1999), 22–4; G.H. Brundtland, ‘Viewpoint’, *European Journal of Public Health*, 15 (2005), 3–5.

105.
R. Rowden, *The Deadly Ideas of Neoliberalism* (New York: Zed Books, 2009); Reubi, *op. cit.* (note 60); L. McGoey, ‘The Philanthropic State’, *Third World Quarterly*, 35 (2014), 109–25.

106.
G.R. Brundtland, ‘Achieving Worldwide Tobacco Control’, *Journal of the American Medical Association*, 284 (2000), 750–1: 751. See also: D. Yach and D. Bettcher, ‘Globalisation of Tobacco Industry Influence and New Global Responses’, *Tobacco Control*, 9 (2000), 206–16; Reynolds and Tansey, *op. cit.* (note 2), 12–13.10.1136/tc.9.2.206PMC174834610841858

107.
Yach and Bettcher, *op. cit.* (note 106), 206.

108.
Yach and Bettcher, *op. cit.* (note 104), 739.

109.
Interview, October 2014. See also Reynolds and Tansey, *op. cit.* (note 2), 66.

110.
CDC, *Global Adult Tobacco Survey* (Atlanta, GA: CDC, 2009).

111.
Yach and Bettcher, *op. cit.* (note 106), 211–2; CDC, GATS Frequently Asked Questions (Atlanta, GA: CDC, 2011), 1.

112.
Reynolds and Tansey, *op. cit.* (note 2), 38–9; Yach, *op. cit.* (note 2).

113.
Bloomberg Philanthropies, *op. cit.* (note 96), 1–4; Rockefeller Foundation, *op. cit.* (note 102).

114.
WHO, *op. cit.* (note 4), 21; Reynolds and Tansey, *op. cit.* (note 2), 40, 47 and 51–3.

115.
Lu *et al.*, *op. cit.* (note 98), v.

116.
Yach, *op. cit.* (note 2); Reynolds and Tansey, *op. cit.* (note 2), 38 and 47.

117.
L.T. Larsen, ‘The Political Impact of Science’, *Science and Public Policy*, 35 (2008), 757–69; WHO, *op. cit.* (note 4), 3.

118.
Reynolds and Tansey, *op. cit.* (note 2), 18–20: 28–30 and 37–8.

119.
See: articles 5.3, 8, 12 and 13 FCTC; Bloomberg Philanthropies, *op. cit.* (note 96), 15–22.

120.
WHO, *op. cit.* (note 96), 56. See also: articles 6, 15 and 17 FCTC; Bloomberg Philanthropies, *op. cit.* (note 96), 23; Reynolds and Tansey, *op. cit.* (note 2), 12 and 23–4.

121.
Reubi, *op. cit.* (note 60), 213.

122.
Reubi, *op. cit.* (note 60). See: Forbes, Frecker and Nostbakken, *op. cit.* (note 46), 483–504; American Cancer Society, *op. cit.* (note 46), 46–50; Slama, *op. cit.* (note 86), 213–40 and 953–62; Lu *et al.*, *op. cit.* (note 98), 697–704; World Bank, *op. cit.* (note 101).

123.
V. Adams, ‘Evidence-based global public health’, in J. Biehl and A. Petryna (eds), *When People Come First* (Princeton, NJ: Princeton University Press, 2013), 54–90; K. Storeng and D. Behague, ‘Evidence-based advocacy and the reconfiguration of rights language in safe motherhood discourse’, in A. Mold and D. Reubi (eds.), *Assembling Health Rights in Global Context* (London: Routledge, 2013), 149–68.

124.
See, for example: Gray and Daube, *op. cit.* (note 32), Appendix 2 on Standardisation of Measurement of Cigarette Smoking Rates; WHO, *Guidelines for the Conduct of Tobacco Smoking Surveys of the General Population* (Geneva: WHO, 1983).

125.
Reubi, *op. cit.* (note 1), 3.

126.
Reynolds and Tansey, *op. cit.* (note 2), 20 and 34; See, for example: R. Peto *et al.*, *Mortality from Smoking in Developed Countries, 1950–2000* (Oxford: Oxford University Press, 1994); R. Peto, C. Zheng-Ming and J. Boreham, ‘Tobacco: The Growing Epidemic’, *Nature Medicine*, 5 (1999), 15–7; R. Peto and A. Lopez ‘Future worldwide health effects of current smoking patterns’, in C.E Koop, C. Pearson and M. Schwarz (eds), *Critical Issues in Global Health* (San Fransisco, CA: Jossey-Bass, 2000), 154–61.

127.
IARC, *Methods for Evaluating Tobacco Control Policies* (Lyon: IARC, 2008); CDC, *op. cit* (note 110).

128.
Interview, April 2013.

129.
IDRC, *op. cit.* (note 103).

130.
Weindling, *op. cit.* (note 24); Bashford, *op. cit.* (note 7); Birn, *op. cit.* (note 24).

131.
Yach, *op. cit.* (note 2).

132.
Interview, January 2015.

133.
Berridge *et al.*, *op. cit.* (note 7).

134.
Birn, *op. cit.* (note 24).

135.
Weindling, *op. cit.* (note 24).

136.
See, for example: Brown *et al.*, *op. cit.* (note 1); Bashford, *op. cit.* (note 7); Bhattacharya, *op. cit.* (note 7).

